# Pseudo-Meigs’ syndrome secondary to breast cancer with ovarian metastasis: a case report and literature review

**DOI:** 10.3389/fonc.2023.1091956

**Published:** 2023-05-08

**Authors:** Xiang-Ying Lin, Xiao-Jun Zhou, Shi-Ping Yang, Jia-Xuan Zheng, Zhao-Jun Li

**Affiliations:** ^1^ Department of Radiation Oncology, Hainan General Hospital (Hainan Affiliated Hospital of Hainan Medical University), Haikou, China; ^2^ Department of Clinical Laboratory, Hainan General Hospital (Hainan Affiliated Hospital of Hainan Medical University), Haikou, China; ^3^ Department of Pathology, Hainan General Hospital (Hainan Affiliated Hospital of Hainan Medical University), Haikou, China

**Keywords:** breast cancer, Pseudo-Meigs’ syndrome, ovarian metastasis, endocrine therapy, oophorectomy

## Abstract

Ovarian metastasis of breast cancer with pseudo-Meigs’ syndrome (PMS) is extremely rare. Only four cases of PMS secondary to breast cancer with ovarian metastasis have been reported to date. In this report, we present the fifth case of PMS caused by ovarian metastasis of breast cancer. On the 2nd of July 2019, a 53-year-old woman presented to our hospital with complaints of abdominal distension, irregular vaginal bleeding, and chest distress. Color Doppler ultrasound examination revealed a mass approximately 109×89 mm in size in the right adnexal area, accompanied by multiple uterine fibroids and a large amount of pelvic and peritoneal effusions. The patient had no common symptoms and showed no signs of breast cancer. The main manifestations were a right ovarian mass, massive hydrothorax, and ascites. Lab workup and imaging revealed raised CA125 (cancer antigen 125) levels and multiple bone metastases. At first the patient was misdiagnosed with ovarian carcinoma. After the rapid disappearance of oophorectomy hydrothorax and ascites, and decreased CA125 levels, from 1,831.8u/ml to normal range. According to the pathology report, breast cancer was finally diagnosed. The patient underwent endocrine therapy (Fulvestrant) and azole treatment after oophorectomy. At the 40-month follow-up, the patient was still alive and doing well.

## 1 Introduction

Ovarian metastasis from breast cancer is extremely rare. Pseudo-Meigs’ syndrome (PMS) secondary to ovarian metastasis is also a rare phenomenon. The uncommon metastatic site and rarity of PMS make ovarian metastasis of breast cancer with PMS extremely rare. To the best of our knowledge, only four cases have been reported worldwide; all of these case reports were reported in Japan (1–4). We reviewed our hospital records, from January 1990 to December 2021, to find all the recorded cases which occurred at our hospital; our search only generated one case.

As early as 1934, Salmon reported two cases of pelvic benign tumor with pleural and peritoneal effusion (5). In 1937, Meigs and Cass reported seven cases of patients who presented with ovarian fibroma with ascites, and pleural effusion, which disappeared after the ovarian fibroma was removed; these cases were clinically established as Meigs’ syndrome (6). According to the literature there are four types of Meigs’ syndrome: thecoma, fibroma, granulosa cell tumor, and Brenner’s tumor. Later, researchers defined PMS according to its tumor histology: other benign or malignant pelvic tumors that cause pleural and peritoneal effusions similar to Meigs’ syndrome (7), including primary malignant tumors, metastases, or other benign tumors of the ovary. The mechanism of hydrothorax and ascites in patients with Meigs’ and PMS remains unclear. Hydrothorax and ascites disappear spontaneously after oophorectomy, and the reason is currently unknown.

The incidence of PMS is very low and it is easily misdiagnosed. PMS is often secondary to digestive tract tumors, while ovarian metastasis of breast cancer is extremely rare. Here, we not only report the first case from China, but also summarize the previous four cases.

## 2 Case representation

**Figure 1:**
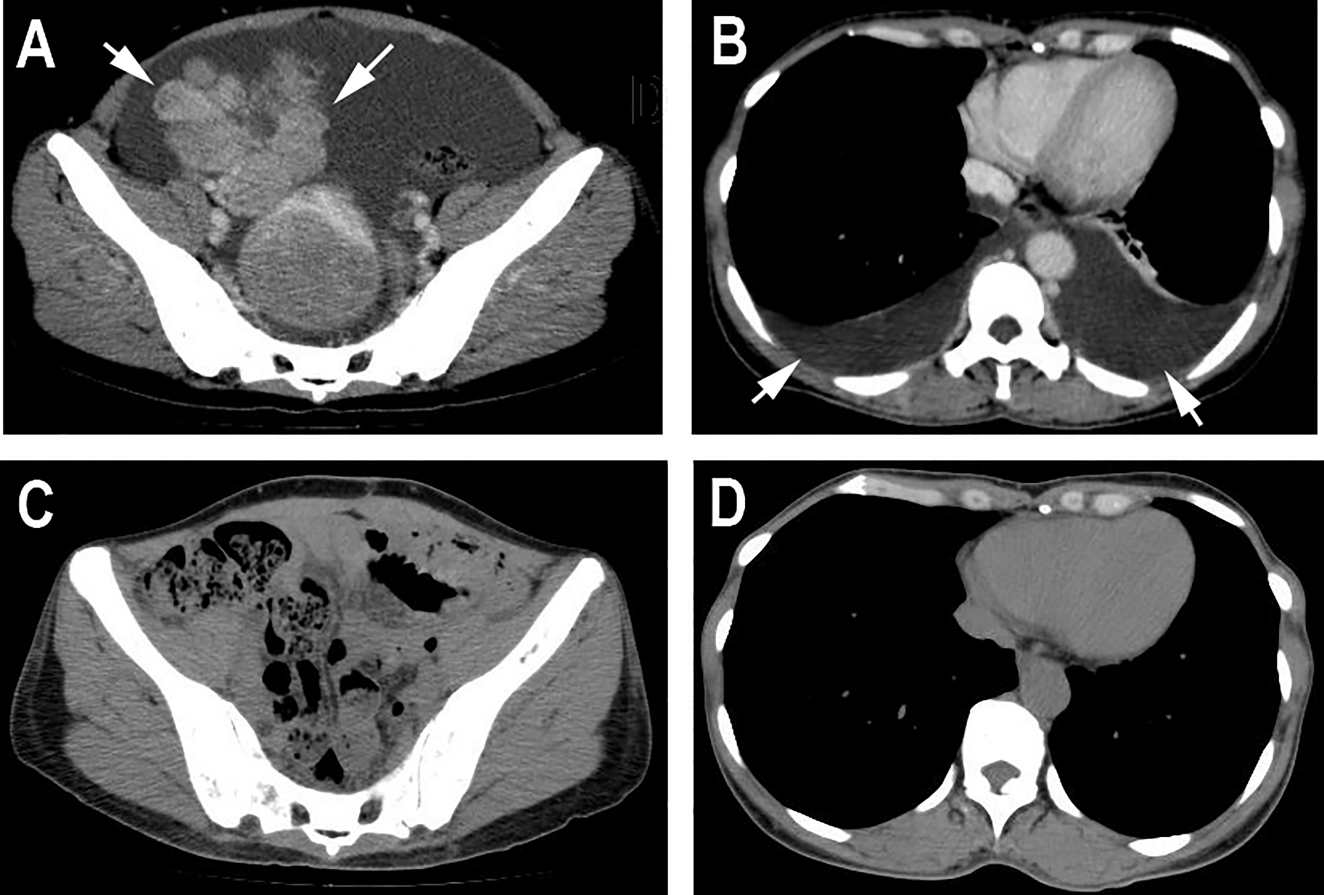
Computed tomography. **(A)** Pelvic CT before the surgery showed a huge mass in the middle and lower abdomen accompanied by ascites. **(B)** Chest CT before the surgery showed bilateral pleural effusion and partial dilatation of the lower lobe of both lungs. **(C, D)** CT after the surgery showed that the huge masses in the pelvis and the bilateral pleural effusion had disappeared.

**Figure 2:**
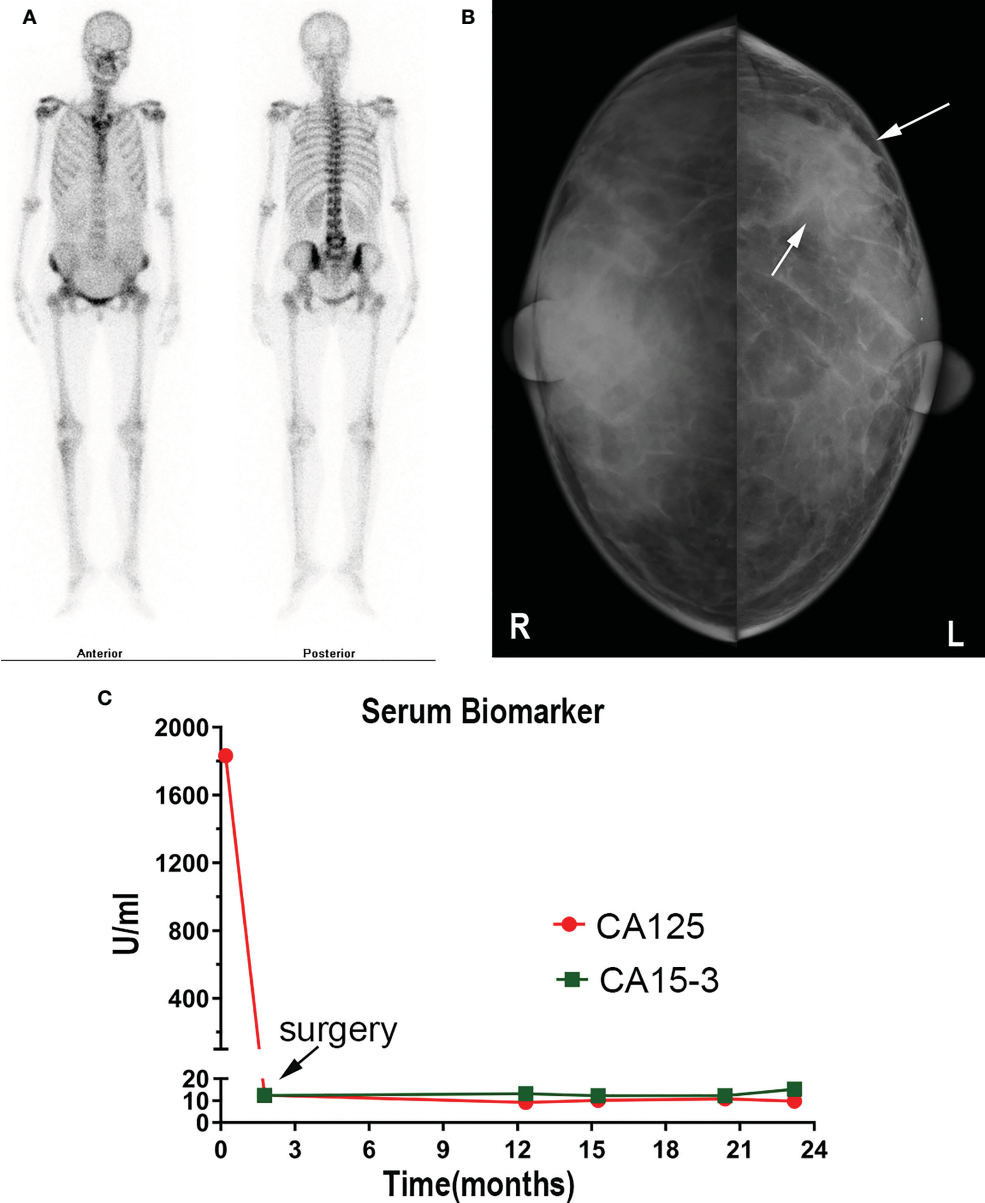
**(A)** SPECT showed abnormal bone metabolism in the left 8^th^ posterior costal vertebra, 4^th^ and 5^th^ lumbar vertebra, and 1st sacral vertebra. **(B)** Mammography showed a dense mass in the left breast. **(C)** CA15-3 and CA 125 level changes are shown.

On the 2nd of July 2019, a 53-year-old woman presented at our hospital with complaints of abdominal distension (for 3 months), irregular vaginal bleeding (for 2 months), and chest distress (for 1 month). Color Doppler ultrasound examination revealed a mass of approximately 109×89 mm in size in the right adnexal area, accompanied by multiple uterine fibroids and a large number of pelvic and peritoneal effusions. The computed tomography (CT) of the pelvic cavity considered that the space occupying lesion in the middle and lower abdomen originated from the right ovary. The chest CT showed moderate effusion in the bilateral pleural cavity and partial dilatation of the lower lobe of both lungs (**Figure 1**). SPECT showed abnormal bone metabolism in the left 8^th^ posterior costal vertebra, the 4^th^ and 5^th^ lumbar vertebra, and the 1^st^ sacral vertebra (**Figure 2A**). Lab work revealed that the tumor marker CA125 (cancer antigen 125) was 1,831.8U/mL.

**Figure 3:**
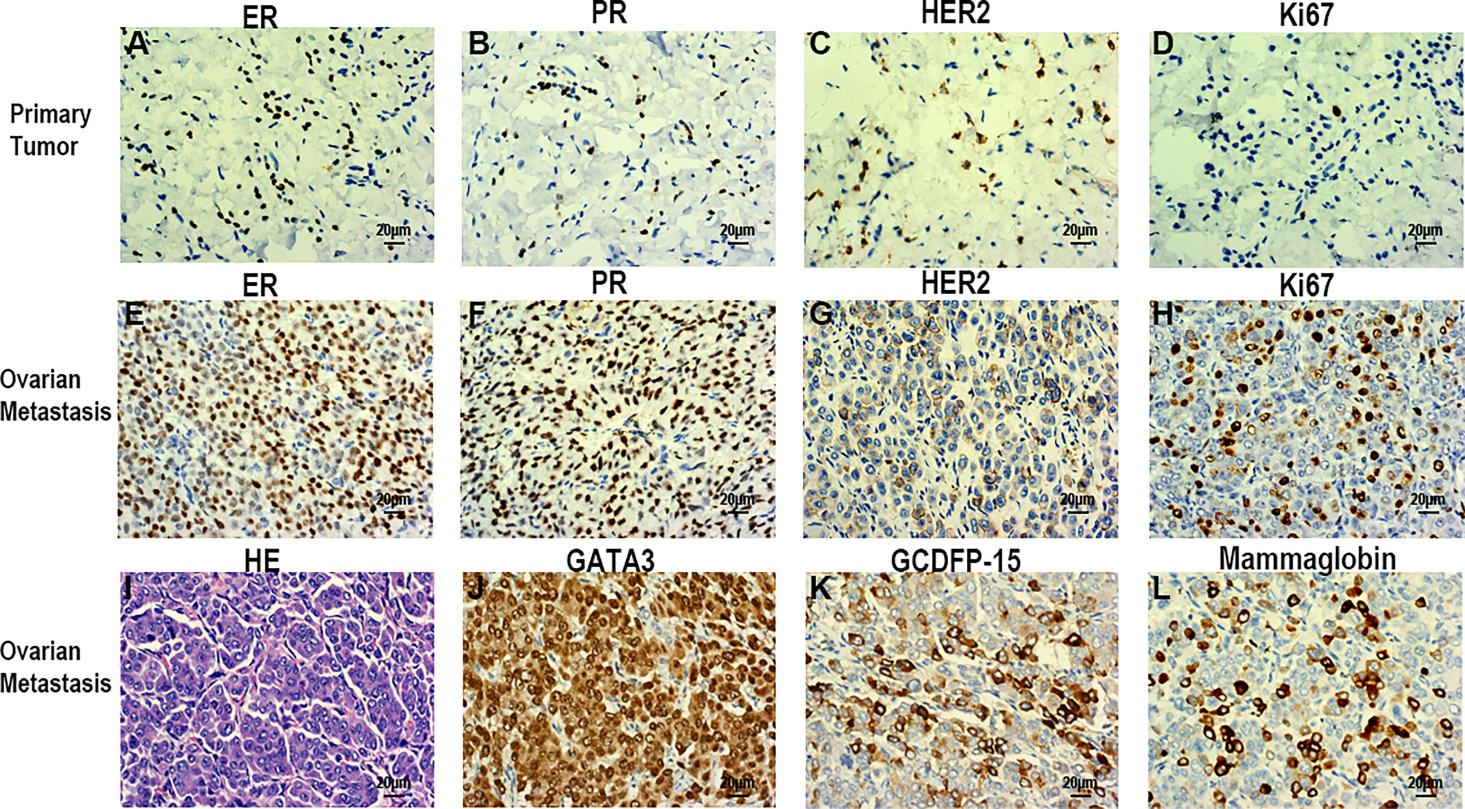
Immunohistochemical examination of breast cancer **(A-D)**. **(A)** ER (+). **(B)** PR (+). **(C)** HER2(+). **(D)** Ki-67(30%). Histopathological and immunohistochemical examination of the ovarian tumor **(E-L)**. **(E)** ER (+). **(F)** PR (+). **(G)** HER2 (+). **(H)** Ki-67 (5%). **(I)** HE staining. **(L)** GATA-3(+). **(K)** GCDFP15(+). **(L)** Mammaglobin (+). Scale bars: 20 µm; magnification, x40.

The patient underwent a comprehensive ovarian cancer staging on July 16, 2019. The surgeons removed the patient’s uterus, bilateral adnexa, bilateral pelvic lymph nodes, abdominal para-aortic lymph nodes, greater omentum, and appendix. During the operation, an irregularly shaped solid multiple nodular mass of approximately 12×10 cm in size was found on the right ovary with soft texture and a ruptured tissue surface. The abdominal cavity was characterized by yellowish-green ascites of 3,500 ml and multiple uterine fibroids. Intraoperative rapid freezing pathology revealed adenocarcinoma of the right adnexa. The pathological report showed right adnexal adenocarcinoma, accompanied by intravascular carcinoma thrombectomy and right fallopian tube invasion. The surgeon re-requested the patient’s medical history and found she had a history of a left breast mass for 2-3 years. She had no discomfort and never went to the hospital for an examination. Combining the immunohistochemical results and clinical history, it was necessary to exclude breast tumor metastasis before considering primary ovarian tumor. Metastatic lymph node cancer was found 12/15: 5/6 left pelvic lymph nodes, 3/5 right pelvic lymph nodes, 2/2 left para-aortic lymph nodes, and 2/2 right para-aortic lymph nodes. Immunohistochemistry tests revealed Ki-67 30%, P53 (+), vim (-), CA125 (-), estrogen receptor (ER) (+), progesterone receptor (PR) (+), a-inhibin (-), Placental alkaline phosphatase (PLAP) (-), S-100 (-), mammaglobin (+), GATA-3(+), and gross cystic disease fluid protein 15 (GCDFP15) (+) (**Figures 3A–D**). Nuclear heterogenous cells were found in ascites. The result of pleural fluid puncture suggested inflammatory exudative lesions. One week after the removal of the ovarian mass, the patient’s pleural and abdominal effusion completely disappeared, the symptoms of abdominal distension and chest distress were relieved, and CA125 levels decreased from 1,831.8 U/ml back to the normal range (**Figure 2C**). Post-surgery, the patient underwent various breast examinations according to pathological indications to trace the primary lesion. Mammography suggested that there was a dense mass near the chest wall in the deep upper outer quadrant of the left breast with the surrounding structural disorder (**Figure 2B**). Breast ultrasound revealed a hypoecho group with a size of 87×10×20 mm in the outer upper quadrant of the left breast, categorized according to the Breast Imaging Reporting and Data System (BI-RADS) as a category 4B. Accompanied by left axillary lymph node enlargement (18×8 mm). The patient underwent a biopsy of the left breast, and the pathology report revealed invasive breast carcinoma. Immunohistochemistry tests revealed ER (+), PR (+), CA125 (-), cytokeratin (CK) (+), CK7 (+), p53 (-), GCDFP15 (+), Ki-67 (5%), and Human epidermal growth factor receptor 2 (HER2) (2+) (**Figures 3E–H**). FISH showed HER2 without amplification. The patient was diagnosed with the following: left breast cancer with ovarian and multiple bone metastases, staging T3N1M1 (ovarian, bone), and Luminal type A. Hormone receptor positive (HR+) patients without visceral crisis, according to NCCN guidelines therapeutic principles, the patient was treated with Fulvestrant and azolephosphonic after the breast cancer diagnosis. At the 40-month follow the patient was doing good and stable.

## 3 Discussion

Ovarian metastases account for 15% of ovarian tumors, mainly from organs such as the gastrointestinal tract (8–10) and the endometrium (11). The ovarian metastasis rate from the breast differs significantly, accounting for approximately 1.8-38% (12–14). Fujii believes that the difference in the incidence of different cancer types could be due to ethnic differences, but the reported difference in ovarian metastasis rate may be more related to the small statistical sample size (1). Nevertheless, less than 10% of patients with breast cancer have evidence of distant metastasis at the time of initial diagnosis (3). Pseudo-Meigs’ syndrome caused by ovarian metastasis of breast cancer is extremely rare, whether it is found at the same time with PMS or heterochronous breast cancer, it is clinically confusing.

Although several theories have been proposed, the etiology of ascites in this clinical syndrome remains unclear. As a first theory, Meigs suggested that the irritation of the peritoneal surface by a hard solid ovarian tumor could stimulate the production of peritoneal fluid (15). A second theory suggests the lymphatics of the tumor (16). A third theory suggests that stromal edema and transudation may occur as a result of a discrepancy between the arterial supply to the large tumor and the venous and lymphatic drainage of the same mass (17). A fourth theory suggests that the excessive production of fluid by the peritoneum leads to ascites (18). The final theory, but probably the most plausible, is that increased capillary permeability and the resultant third-space fluid shift occur due to increased levels of inflammatory cytokines and vascular endothelial growth factor (VEGF) (19).

The main clinical challenge of PMS is that it can easily be misdiagnosed as carcinomatous peritonitis or pleurisy, but the cytological results of pleural and ascites effusion in Meigs’ syndrome/PMS should be negative. The conditions of patients with PMS are often confused with terminal stage malignant diseases, for which curative surgical treatment is not an option and surgery is merely introduced as a palliative approach.

**Table 1** summarizes the previous four cases as well as the case presented in this report. The four patients were aged 34, 50, 54, and 49 years old. Our patient was 53 years old at the time of the diagnosis and treatment. Two of the previous four cases were metachronous, accompanied with ascites and pleural effusion, with elevated CA15-3 (cancer antigen 15-3) levels. Two of the previous four cases presented with both pleural effusion and elevated CA125 levels. Our case presented with both ascites and pleural effusion, as well as elevated CA125 levels. Oophorectomy was performed in all of the five cases. CA125 levels was significantly elevated in this case, while CA15-3 was only elevated in some patients and normal in others. Thus, the tumor origin may be difficult to determine by merely relying on these serum tumor markers. Primary tumor differentiation ultimately depends on the diagnosis of the pathological specimens. GATA-3, GCDFP-15, and Mammaglobin are important immunohistochemical indexes that indicate the origin of breast cancer (20). Compared with the ER, PR, HER2, Ki67 and other immunohistochemical indexes of primary breast and ovarian metastases, there may be inconsistencies between metastatic lesions and primary lesions (**Figure 3**).

**Table 1:** The clinical characteristic and survival of all the cases.

Author (year)	Age (years)	Onset (interval/months)	CA125 (U/ml)	CA15-3 (U/ml)	ER(breast/ovary)	PR(breast/ovary)	HER-2(breast/ovary)	Metastasis of other organs	Primary tumor resection	oophorectomy	CT/RT	ET/anti-Her2	Follow-up
Fujii (2006) (1)	34	M (52)	ND	High	-/+	-/+	ND/+	Uterus, abdominal cavity, sigmoid colon and omentum	Yes	Yes	Capecitabine, mitomycin-C +methotrexate +mitoxantrone hydrochloride	Aromatase inhibitor, toremifene citrate and trastuzumab	Dead after 2.2 years of oophorectomy
Kawakubo (2010) (2)	50	S	4488	10.5	+/+	-/-	-/-	Bone and liver	No	Yes	Docetaxel	ND	Dead after 4 months of oophorectomy
Naito (2012) (4)	54	M (69 )	ND	High	+/+	+/+	-/-	ND	Yes	Yes	Paclitaxel and carboplatin	Aromatase inhibitor	40 months (stable)
Akizawa (2021) (3)	49	S	692	93.9	+(left) &-(right)/+	+(left) &-(right)/+	-(left) &-(right)/-	Bone	Yes	Yes	Capecitabine	Palbociclib, letrozole	17 months (stable)
present case	53	S	1831.8	12.36	+/+	+/+	-/-	Bone	No	Yes	No	Fulvestrant	40 months(stable)

M, metachronous; S, synchronousm; ER, estrogen receptor; PR, progesterone receptor; ND, no document; CT, chemotherapy; RT, radiotherapy.

Ovarian metastases are usually large, and surgery to reduce the tumor load may be helpful (1–4, 21). In all the reported cases, the hydrothorax and ascites rapidly disappeared after resection of the ovarian metastases. In contrast, the resection of the primary breast tumor is currently a big debate. PMS caused by breast cancer is often accompanied by distant metastasis. Three of the previous four cases had multiple metastases of abdominal organs, liver and bone, respectively. But one of the previous four cases had remained stable for 40 months (**Table 1**).

In our reported case, there was only bone metastases but no signs of visceral metastases. The effective disease management and treatment approach was determined using the NCCN guidelines. This patient was Luminal type A and had no visceral crisis, so she was given endocrine therapy with Fulvestrant. Under these circumstances, the primary question to answer is whether primary breast tumor resection should be carried out? To answer this primary question, surgeons always work in coordination with a multidisciplinary team. Which was the case in our report, the case was discussed by the multidisciplinary team. The breast surgeon was of the opinion that the primary breast lesion should not be removed. After surgery, there was no recurrence of hydrothorax and ascites, no new metastases, and at the 40-month follow up in November 2022, the patient’s KPS score was 90.

## 4 Conclusion

Cases of ovarian metastasis of breast cancer with PMS is extremely rare. To the best of our knowledge, this is only the fifth case. Our case report demonstrates that curative surgery for PMS secondary to breast cancer with ovarian metastasis resulted in a good KPS score and could be possible. At the 40-month follow-up, dated November 2022, the patient was still alive and doing well, with no indication of decline.

## Data availability statement

The original contributions presented in the study are included in the article/**Supplementary Material**. Further inquiries can be directed to the corresponding author.

## Ethics statement

Written informed consent was obtained from the individual(s) for the publication of any potentially identifiable images or data included in this article.

## Author contributions

Data acquisition: Z-JL, X-JZ and J-XZ. Data analysis and interpretation: Z-JL, S-PY. Radiological analysis of ultrasound and CT images: Z-JL, X-YL. Manuscript preparation: X-YL and Z-JL. S-PY contributed the idea of this article writing. All authors contributed to the article and approved the submitted version.

## Supplementary material

The Supplementary Material for this article can be found onlineat: https://www.frontiersin.org/articles/10.3389/fonc.2023.1091956/full#supplementary-material
