# Supplementary figures and images for: Pseudo-Meigs’ syndrome secondary to breast cancer with ovarian metastasis: a case report and literature review
Source: Front Oncol. 2023 May 8;13:1091956. doi: 10.3389/fonc.2023.1091956 (PMC10200991; doi:10.3389/fonc.2023.1091956)

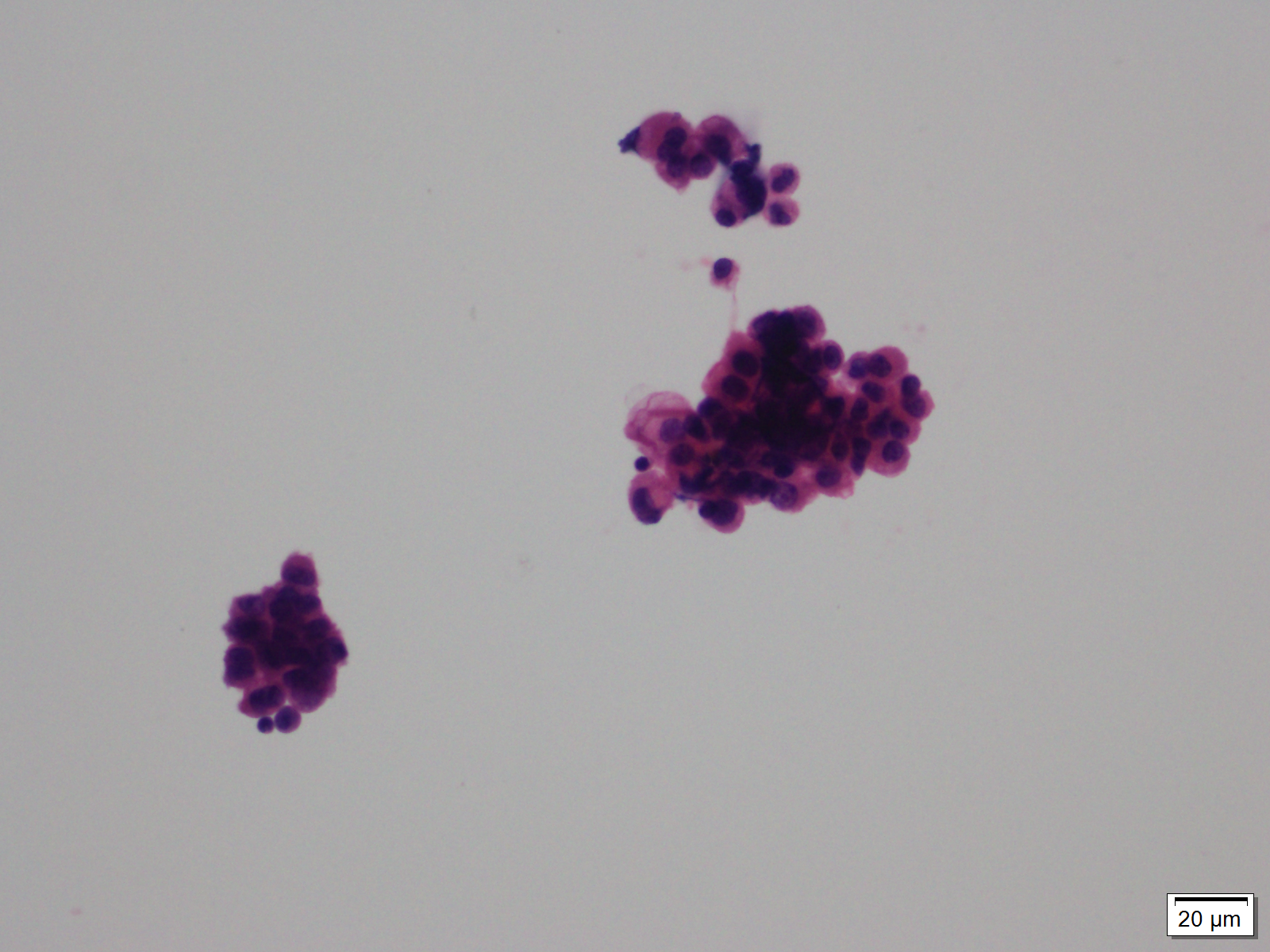

Supplement: Supplementary file 1 [file Image_1.tif]
